# P094 Melkersson–Rosenthal syndrome in association with primary Sjögren’s syndrome: a case report

**DOI:** 10.1093/rap/rkag113.124

**Published:** 2026-11-03

**Authors:** Conor Gormley, Aine Gorman

**Affiliations:** MRH Tullamore, Tullamore, Ireland; MRH Tullamore, Tullamore, Ireland

## Abstract

**Introduction:**

Melkersson–Rosenthal syndrome (MRS) is a rare neuro-mucocutaneous disorder characterised with the triad of recurrent facial paralysis, orofacial oedema and tongue fissuring. The symptoms very rarely present simultaneously (8-18%) and it thus can be a challenging diagnosis. It has been associated with several chronic inflammatory and autoimmune conditions, including sarcoidosis and inflammatory bowel disease. To the best of our knowledge this is the first reported case of MRS associated with Sjögren’s syndrome.

**Case description:**

A 52-year-old woman was referred to Dermatology in May 2024 with an 18-month history of a slowly progressive, infiltrated erythematous, non-pruritic, non tender plaque on the right side of her face. Initial punch biopsy showed a dermal infiltrate composed of lymphocytes, plasma cells and histiocytes without granulomas, and serum investigations revealed elevated ACE (83, normal <65) and markedly positive anti-Ro antibodies (170, normal <7), prompting Rheumatology referral. Incisional biopsy in July 2024 again demonstrated a non-granulomatous inflammatory infiltrate.

In the Rheumatology clinic in September 2024, she described three self-limiting episodes of right-sided facial palsy over the preceding year, each lasting four to five days and accompanied by ipsilateral facial and lip swelling around the plaque. The patient had no other medical background and was taking no regular medications. She denied limb rash, chest pain or dyspnoea, and chest radiography showed no hilar lymphadenopathy, arguing against pulmonary sarcoidosis. She also described a 3–4 year history of dry eyes and dry mouth; in the context of strongly positive anti-Ro antibodies, a diagnosis of primary Sjögren’s syndrome was made. MRI and MRA of the brain in November 2024 were unremarkable excluding any intracranial pathology.

At combined Dermatology–Rheumatology review in December 2024, examination revealed horizontal fissuring of the tongue (lingua plicata) together with a history of recurrent orofacial swelling and facial palsy, fulfilling the clinical triad of Melkersson–Rosenthal syndrome (MRS).

She was commenced on azathioprine 75 mg once daily (after not tolerating hydroxychloroquine) in January 2025, with subsequent escalation to 150 mg daily in May 2025, leading to a marked reduction in facial swelling and no further facial palsy episodes, although residual skin laxity under the right eye persisted. A CT thorax and liver in February 2025 showed no thoracic lymphadenopathy.

**Discussion:**

Melkersson–Rosenthal syndrome (MRS) is a rare neuro-mucocutaneous disorder characterised by recurrent orofacial swelling, relapsing facial nerve palsy and fissured tongue, although the full triad is often not present at presentation. In this case, the diagnosis was initially challenging because the patient presented with a chronic unilateral infiltrated facial plaque and non-specific histology on repeated biopsy.

The differential diagnosis included sarcoidosis, chronic infection, angioedema and other granulomatous or inflammatory conditions. Although her serum ACE was mildly elevated, there was no radiological evidence of thoracic disease and no histological support for sarcoidosis. The history of recurrent self-limiting facial palsy, associated facial swelling and later identification of fissured tongue were more in keeping with MRS.

An important aspect of this case is the coexistence of newly diagnosed Sjögren’s syndrome, supported by a prolonged (several years) history of sicca symptoms and strongly positive anti-Ro antibodies. MRS has recognised associations with inflammatory conditions including sarcoidosis and inflammatory bowel disease, suggesting an immune-mediated basis in at least a subset of patients. However, Sjögren’s syndrome has not, to current knowledge, been previously described in association with MRS in the literature, making this a likely index case. The good response to azathioprine after intolerance of hydroxychloroquine further supports an inflammatory or autoimmune driver in this presentation.

**Key learning points:**

• Melkersson–Rosenthal syndrome should be considered in patients with recurrent unilateral facial swelling and episodic facial palsy, even when biopsy findings are non-specific.

• The full clinical triad may emerge only over time, so repeated multidisciplinary assessment can be essential for diagnosis.

• Melkersson–Rosenthal syndrome may improve when the underlying inflammatory or autoimmune disease is treated, highlighting the importance of looking for and managing systemic associations.

